# Educational inequalities in mortality associated with rheumatoid arthritis and other musculoskeletal disorders in Sweden

**DOI:** 10.1186/s12891-019-2465-8

**Published:** 2019-02-18

**Authors:** Aliasghar A. Kiadaliri, Ingemar F. Petersson, Martin Englund

**Affiliations:** 1 Department of Clinical Sciences Lund, Lund University, Faculty of Medicine, Orthopaedics, Skåne University Hospital, Clinical Epidemiology Unit, Remissgatan 4, SE-221 85 Lund, Sweden; 2 Skåne University Hospital, Lund, Sweden; 3 Clinical Epidemiology Research and Training Unit, Boston University School of Medicine, Boston, MA USA

**Keywords:** Educational inequality, Musculoskeletal disorders, Rheumatoid arthritis, Multiple-cause-of death, Sweden

## Abstract

**Background:**

Musculoskeletal (MSK) disorders are less likely to be reported as an underlying cause of death (UCD) and since cause of death studies are generally limited to the UCD, little is known about socioeconomic inequalities in MSK disorders as cause of death in the general population. Using multiple-cause-of-death data, we aimed to quantify and compare educational inequalities in musculoskeletal (MSK) disorders- with non-MSK disorders-related mortality.

**Methods:**

All residents aged 30–99 years in the Skåne region, Sweden, during 1998–2013 (*n* = 999,148) were followed until their 100th birthday, death, relocation outside Skåne, or end of 2014. We identified any mention of rheumatoid arthritis (RA) or other MSK disorders on death certificates using multiple-cause-of-death data. We retrieved and linked individual-level data from Statistics Sweden on highest level of education. We used Cox regression and additive hazards models with age as time-scale adjusted for sex, marital status, and country of birth to calculate slope and relative indices of inequality (SII/RII).

**Results:**

During a mean follow-up of 12.2 years, there were 1407 (0.8% of all deaths) and 3725 (2.1% of all deaths) death certificates with mention of RA and other MSK disorders, respectively, and 171,798 death certificates without any mention of a MSK disorder. Age-standardized RA mortality rate was 2.2 (95% confidence interval [CI]: 2.0–2.8) times greater in people with 0–9 years of education compared with those with > 12 years of education. Corresponding figure for other MSK disorders was 1.5 (95% CI: 1.4–1.6). Both RIIs and SIIs revealed statistically significant educational inequalities in RA/other MSK disorders mortality favouring high-educated people. The RIIs of MSK disorders-related deaths were generally greater than non-MSK disorders-related deaths.

**Conclusion:**

We found substantial educational inequality in mortality from MSK disorders. Further research is needed to investigate underlying pathways driving these inequalities.

**Electronic supplementary material:**

The online version of this article (10.1186/s12891-019-2465-8) contains supplementary material, which is available to authorized users.

## Background

Low socioeconomic status (SES) is often associated with worse health outcomes in patients with musculoskeletal (MSK) disorders including rheumatoid arthritis (RA) [1–3]. SES is also an important predictor of mortality in people with MSK disorders [1, 3–7]. However, little is known about socioeconomic inequalities in RA and other MSK disorders as *cause of death*. This is mainly because cause of death studies are generally limited to the underlying cause of death (UCD) and MSK disorders are less likely to be reported as an UCD particularly among older people suffering from comorbidities [8]. This limitation can be dealt with using multiple-cause-of-death (MCD) data, i.e. examining *all* diseases mentioned on a death certificate [8, 9]. Thus, in the current study, we aimed to quantify the absolute and relative educational inequalities in mortality related to RA and other MSK disorders *as cause of death* at the population level using the MCD data in Sweden and compare these with educational inequalities in non-MSK related-mortality.

## Methods

### Setting and data

We conducted a longitudinal register-based cohort study of the inhabitants in the southernmost region of Sweden, Skåne, with a population of about 1.3 million (13.2% of the Sweden’s population) in 2014. We identified all residents of the region aged 30–99 years from 1st January 1998 to 31st December 2013 using the Swedish Population Register. This register contains data on sex, births, deaths, and residential address. We obtained the individual-level data on all death certificates issued in the region during 1998–2014 from the Swedish National Board of Health and Welfare’s Cause of Death Register (http://www.socialstyrelsen.se/). We extracted the following data from death certificates: date of death, the UCD, and up to 20 additional causes of death according to the International Classification of Diseases, 10th revision (ICD-10).

### Socioeconomic status

We obtained data on education from the longitudinal integration database for health insurance and labour market studies (LISA). The database LISA presently holds annual registers since 1990 and includes all individuals 16 years of age and older that were registered in Sweden as of December 31 for each year. The database integrates existing data from the labour market, educational and social sectors and is updated each year with a new annual register (https://www.scb.se). We divided the highest level of attained education into three categories: “low” (0–9 years of education), “medium” (10–12 years of education), and “high” (> 12 years of education). We also obtained the data on marital status (never married, previously married, and married) and country of birth as potential confounders from the LISA. All registers were linked on the individual level using the personal identification number assigned to all residents in Sweden.

### Outcome and follow-up

For cause-of-death attribution, we used “any mention” approach where a death certificate with any mention of the disease of interest (RA and other MSK disorders in our study) on any part of the death certificate (i.e., underlying or contributing cause) is considered as an event. We identified RA- and other MSK disorders-related death as a death certificate with any mention of RA (ICD-10 codes: M05-M06) or other MSK disorders (ICD-10 codes: M00-M99 excluding M05-M06). Non-MSK disorders deaths were defined as death certificates without any mention of RA or other MSK disorders. Each subject’s follow-up started at January 1, 1998 (the beginning of the study period), or January 1 of the year of becoming a resident of the Skåne, or his/her 30th birthday, whichever occurred last. All subjects were followed until their 100th birthday, death, relocation outside Skåne, or end of 2014, whichever occurred first.

### Statistical analysis

For each level of education, we computed directly the age-standardized mortality rate per 100,000 person-years using the Sweden population in the year 2000 as standard population. In computing the person-years at risk, we accounted for changes in individual’s age during the follow-up. We measured the absolute educational inequality using the slope index of inequality (SII) and relative educational inequality using hazard ratio (HR) and relative index of inequality (RII). To calculate SII and RII, each level of education was assigned a fractional rank based on the mean proportion of the population with a higher level of education [10]. Therefore fractional rank is a continuous variable ranging from 0 (the highest education) to 1 (the lowest education).

We used cause-specific Cox proportional hazard model with age as time-scale, accounting for late entry, to estimate the HR (using the high level of education as the reference category) and the RII [10, 11]. We estimated RII by including the fractional rank in the model and the exponential of its coefficient, which represent the ratio of mortality rates between the two extremes of the educational hierarchy. We used Schoenfeld’s residuals plot to assess the proportional hazards assumption and since the assumption was not met, we fitted models separately for three age strata: 30—69 years, 70—79 years, and 80 years or older. As we used age as the time scale, these age strata refer to attained age. For instance, if someone entered the study at age 68 and was followed until age 82, s/he contributed for 2 years to the age stratum 30—69 years, for 10 years to the age stratum 70—79 years, and for 2 years to the age stratum 80+ years. The SII was estimated by fitting an additive hazard model with age as time-scale and fractional rank as predictor (its coefficient gives an estimate of SII representing the absolute difference in mortality rates between the two extremes of the educational hierarchy) [10]. All models were adjusted for sex, marital status, and country of birth. Analyses were performed using Stata version 13 and R version 3.2.5.

## Results

We identified 1,091,548 people aged 30–99 years who were registered as the Skåne resident at least once between 1998 and 2013. Of these, 21,903 (2.0%) were excluded with missing follow up data (e.g., people who were registered only once in the register with no further information on place of residence or vital status), 70,409 (6.6%) had missing data on level of education, and 88 with missing information on country of birth. A total of 999,148 persons with over 12 million person-years of follow-up were included in the analyses (Table 1).

**Table 1:** Sociodemographic characteristics, number of deaths, and person-years follow-up by education group

	Education			
	Low	Medium	High	Missing
Number of people, n	288,307	406,854	304,075	70,409
Women,%	51.8	49.2	51.9	52.8
Age at entry, %				
30–49	34.6	66.1	75.6	52.3
50–64	26.9	20.6	17.4	11.0
65–79	29.0	11.0	6.1	9.4
80+	9.5	2.3	0.9	27.3
Marital status, %				
Never married	19.6	33.1	38.2	23.1
Previously married	26.9	16.2	10.5	29.1
Married	53.5	50.7	51.3	46.4
Missing	0.0	0.0	0.0	1.4
Born in Sweden, %	83.6	83.7	79.7	31.5
Deaths, number				
Rheumatoid arthritis	908	397	102	105
Other MSK disorders	2305	1097	323	477
Non-MSK disorders	99,340	53,243	19,217	22,507
Person-years follow-up	3,598,592	5,200,412	3,404,203	451,261

*MSK* musculoskeletal

During a mean follow-up of 12.2 years, there were 1407 (0.8% of all deaths) and 3736 (2.1% of all deaths) death certificates with mention of RA and other MSK disorders, respectively, and 171,798 death certificates without any mention of a MSK disorder. Among death certificates that mention RA and other MSK disorders, these were recorded as underlying cause of death on 23.4 and 18.0% of death certificates, respectively (Additional file 1: Table S1 and Additional file 2: Table S2). The diseases of the circulatory system (ICD-10 codes: I00-I99) were the leading cause of death mentioned on death certificates related to RA and other MSK disorders.

Women had higher age-standardized mortality rates for RA/other MSK disorders compared with men, and RA mortality rate among women with high education was higher than men with low education ((Additional file 3: Table S3). For both RA and other MSK disorders, age-standardized mortality rates were higher among people with low education compared with high education (rate ratio [95% CI] of 2.2 [2.0–2.4] for RA and 1.5 [1.4–1.6] for other MSK disorders).

The absolute inequality measured by SII ranged from 2 (95% CI: 1–4) to 38 (17–60) RA deaths per 100,000 person-years in age groups 30–69 and 80+ years, respectively (Table 2). The SII for mortality associated with other MSK disorders increased from 6 (95% CI: 5–8) in the age group 30–69 years to 61 (19–103) in the age group 80+ years per 100,000 person-years. Contribution of inequalities in RA deaths to inequalities in deaths associated with MSK disorders (measured as SII for RA divided by sum of SII for RA and other MSK disorders) were 27, 47, and 39% in the age groups 30–69, 70–79, and ≥ 80 years, respectively, with higher proportions among women than men.

**Table 2:** Slope index of inequality (per 100,000 person-years) in deaths from rheumatoid arthritis/other musculoskeletal disorders

	30–69 years			70–79 years			80+ years		
	All	Men	Women	All	Men	Women	All	Men	Women
Rheumatoid arthritis	**2.4(1.4, 3.5)**	**1.4(0.2, 2.5)**	**3.5(1.7, 5.4)**	**27.9(17.8, 38.1)**	**17.4(6.7, 28.0)**	**38.7(22.1, 55.3)**	**38.3(16.6, 60.0)**	**34.0(6.6, 61.5)**	**44.7(12.5, 76.8)**
Other MSK disorders	**6.4(4.8, 8.1)**	**4.7(2.6, 6.9)**	**8.1(5.6, 10.7)**	**31.6(17.6, 45.6)**	**39.2(19.7, 58.7)**	**22.4(2.6, 42.1)**	**60.6(18.6, 102.6)**	**106.8(48.0, 165.6)**	31.8(−26.6, 90.3)

*MSK* musculoskeletal. Models were adjusted for sex, marital status, and place of born (Nordic vs. non-Nordic)

Values in parentheses display 95% confidence interval

Statistically significant results (*p*<0.05) are in bold

Both HRs and RIIs were statistically significantly higher than 1 indicating that mortality associated with RA and other MSK disorders were higher among those with lower education across all age strata (Table 3). Compared with non-MSK disorders-related mortality, the magnitude of RIIs were generally greater for MSK disorders-related mortality. For both RA and other MSK disorders, the RII declined gradually with age and this was more profound among women.

**Table 3:** The relative measures of educational inequality in deaths with/without mention of musculoskeletal disorders

	Rheumatoid arthritis			Other MSK disorders			No mention of MSK disorders		
	Hazard ratio ^a^		RII	Hazard ratio ^a^		RII	Hazard ratio ^a^		RII
	Low	Medium		Low	Medium		Low	Medium	
30–69 years									
All	**2.4 (1.6, 3.7)**	**1.7 (1.1, 2.6)**	**3.4 (1.9, 5.9)**	**2.8 (2.1, 3.7)**	**2.1 (1.5, 2.8)**	**3.8 (2.6, 5.3)**	**1.9 (1.8, 1.9)**	**1.5 (1.4, 1.5)**	**2.4 (2.3, 2.5)**
Men	**2.1 (1.0, 4.7)**	**1.3 (0.5, 2.9)**	**3.2 (1.1, 9.7)**	**2.4 (1.6, 3.7)**	**2.2 (1.4, 3.4)**	**2.7 (1.7, 4.4)**	**1.9 (1.8, 2.0)**	**1.5 (1.5, 1.6)**	**2.3 (2.2, 2.4)**
Women	**2.6 (1.6, 4.4)**	**1.9 (1.1, 3.2)**	**3.7 (1.9, 7.1)**	**3.2 (2.2, 4.7)**	**1.9 (1.3, 2.9)**	**5.1 (3.1, 8.5)**	**1.9 (1.8, 2.0)**	**1.4 (1.4, 1.5)**	**2.5 (2.3, 2.6)**
70–79 years									
All	**2.2 (1.6, 3.2)**	**1.6 (1.1, 2.3)**	**2.7 (1.9, 3.9)**	**1.7 (1.3, 2.1)**	**1.4 (1.1, 1.8)**	**1.9 (1.4, 2.5)**	**1.5 (1.4, 1.5)**	**1.3 (1.2, 1.3)**	**1.6 (1.6, 1.7)**
Men	**2.8 (1.3, 5.8)**	2.0 (0.9, 4.3)	**3.3 (1.5, 7.0)**	**2.0 (1.4, 2.9)**	**1.6 (1.1, 2.4)**	**2.3 (1.5, 3.5)**	**1.4 (1.4, 1.5)**	**1.2 (1.2, 1.3)**	**1.6 (1.5, 1.7)**
Women	**2.1 (1.4, 3.1)**	**1.5 (1.0, 2.3)**	**2.6 (1.7, 4.0)**	**1.4 (1.0, 1.9)**	1.2 (0.8, 1.7)	**1.5 (1.0, 2.2)**	**1.5 (1.4, 1.6)**	**1.3 (1.2, 1.3)**	**1.7 (1.6, 1.8)**
80+ years									
All	**1.8 (1.3, 2.5)**	**1.6 (1.1, 2.2)**	**1.7 (1.2, 2.3)**	**1.3 (1.1, 1.5)**	**1.2 (1.0, 1.4)**	**1.3 (1.1, 1.5)**	**1.3 (1.2, 1.3)**	**1.1 (1.1, 1.2)**	**1.4 (1.3, 1.4)**
Men	**2.1 (1.1, 3.8)**	1.7 (0.9, 3.2)	**2.1 (1.1, 3.9)**	**1.7 (1.3, 2.2)**	**1.5 (1.2, 2.0)**	**1.6 (1.2, 2.2)**	**1.2 (1.2, 1.3)**	**1.1 (1.1, 1.1)**	**1.3 (1.3, 1.4)**
Women	**1.8 (1.2, 2.6)**	**1.6 (1.1, 2.3)**	**1.6 (1.1, 2.2)**	1.1 (0.9, 1.3)	1.1 (0.9, 1.3)	1.1 (0.9, 1.4)	**1.3 (1.3, 1.4)**	**1.2 (1.2, 1.2)**	**1.4 (1.3, 1.4)**

*RII* relative index of inequality, *MSK* musculoskeletal. Models were adjusted for sex, marital status, and place of born (Nordic vs. non-Nordic)

^a^ High education as reference group

Values in parentheses display 95% confidence interval

Statistically significant results (*p*<0.05) are in bold

## Discussion

In a large population-based study using a cohort approach, we found an inverse association between level of education and mortality associated with RA as well as other MSK disorders. The rates of RA/other MSK disorders mortality were generally 2–3 times higher in people with the lowest versus the highest position on the education scale. These disparities varied by age and sex. In addition, the relative educational inequalities in MSK disorders-related mortality appear to be generally greater compared with non-MSK disorders-related deaths.

To our best knowledge, only two previous studies have investigated the association between SES and MSK disorders as *cause of death* in the general population [6, 9]. While one of these [6] did not have a longitudinal design, included people aged 25–64 years, and was limited to deaths from systemic lupus erythematosus, the other one [9] had a similar design as ours and used the RII to measure educational inequality in deaths associated with MSK disorders. However, it included only men, had about 4 years shorter follow-up, and used deaths associated with MSK disorders merely as an example without further discussing of the findings. Nevertheless, our study corroborate and extend the findings that the rate of deaths from MSK disorders among people with low education is greater than those with high level of education.

The educational inequalities in mortality associated with MSK disorders that we found may likely be explained by more frequent or higher level of risk factors (e.g. smoking, obesity, physical inactivity) in those with lower SES, and thus often higher occurrence of MSK disorders and/or more severe MSK disease when present. Further, more hazardous work environment, more frequent or severe comorbidities including cardiovascular and respiratory diseases, lower treatment adherence, self-efficacy and coping mechanisms in people with low education may also contribute [3, 7, 12, 13]. Moreover, despite universal healthcare access in Sweden, we cannot rule out differences in healthcare utilization [14, 15].

Our results showed that the magnitude of relative inequality was greater in deaths with mention of MSK disorders compared with those with no mention of MSK disorders. Furthermore, we assessed the relative educational inequalities in fracture-related mortality in this sample in a previous study [16] and found smaller RII compared with MSK disorders-related mortality. While the reasons for these differences are not obvious and warrant further investigation, this result may reflect larger educational gradient in prevalence of MSK disorders risk factors than in prevalence of other causes risk factors. It should be noted that we pooled all other causes together and hence our finding might not generalizable to specific-causes of death (e.g., neoplasms, cardiovascular diseases, etc.).

The greater absolute educational inequality in RA-related mortality in women than in men might be partially explained by higher RA-related mortality among women. However, while women had higher mortality rate of other MSK disorders, among people aged ≥70 years the absolute educational inequality in other MSK disorders-related mortality was larger in men. The similar finding was found for the relative educational inequalities in both RA- and other MSK disorders-related mortality (i.e., higher RIIs in men than in women among those aged ≥70 years). These findings might reflect larger educational inequalities in prevalence of MSK risk factors, in management of MSK disorders, and in use of health care in older men compared with older women.

Our main strengths include the longitudinal design with a large cohort of the general population followed over 12 years and the use of MCD data to define deaths from MSK disorders. However, despite using MCD data, underreporting and diagnostic inaccuracy of MSK disorders on death certificates is a source of concern. In particular, if quality of cause of death data vary by education, then our estimates would be biased. However, a previous study reported no educational differences in the use of ill-defined causes of death in Sweden [17]. Due to the lack of data, we did not control for several important confounders of the education-MSK mortality association (e.g., early life socioeconomic and health status, cognitive ability). Due to lack of data, we were also unable to further explain the observed educational inequality in mortality associated with MSK disorders, e.g. lifestyle risk factors, obesity level, adherence to treatment, etc.

## Conclusion

In one of the first efforts to assess educational inequality in deaths attributable to MSK disorders including RA, our results suggest that low level of education is associated with higher mortality. We also found that there were greater relative educational inequalities in MSK disorders-related mortality compared with non-MSK disorders-related mortality which warrant further investigation. We suggest that education should be considered in implementing preventive strategies targeting MSK disorders risk factors.

## Additional files

Additional file 1: **Table S1.** Contributing causes of death when rheumatoid arthritis/other musculoskeletal disorders were recorded as the underlying cause of death. The Table displays the distribution of contributing causes of death on death certificates that recorded rheumatoid arthritis or other musculoskeletal disorders as underlying cause of death. (DOCX 12 kb)

Additional file 2: **Table S2.** Underlying causes of death when rheumatoid arthritis/other musculoskeletal disorders were recorded as contributing cause of death. The Table shows the distribution of underlying causes of death on death certificates that recorded rheumatoid arthritis or other musculoskeletal disorders as contributing cause of death. (DOCX 12 kb)

Additional file 3: **Table S3.** Age-standardized mortality rate of rheumatoid arthritis and other musculoskeletal disorders by education group and sex. The Table reports age-standardized mortality rate related to rheumatoid arthritis and other musculoskeletal disorders and low to high education mortality rate ratio, stratified by sex. (DOCX 12 kb)

## A**bbreviations**

CI: Confidence interval
HR: Hazard ratio
MCD: Multiple cause of death
MSK: Musculoskeletal
RA: Rheumatoid arthritis
RII: Relative index of inequality
SES: Socioeconomic status
SII: Slope index of inequality
UCD: Underlying cause of death
