# Supplementary material for: Educational inequalities in mortality associated with rheumatoid arthritis and other musculoskeletal disorders in Sweden
Source: BMC Musculoskelet Disord. 2019 Feb 18;20:83. doi: 10.1186/s12891-019-2465-8 (PMC6379941; doi:10.1186/s12891-019-2465-8)
Supplement: Supplementary file 2 — Table S2. Underlying causes of death when rheumatoid arthritis/other musculoskeletal disorders were recorded as contributing cause of death. The Table shows the distribution of underlying causes of death on death certificates that recorded rheumatoid arthritis or other musculoskeletal disorders as contributing cause of death. (DOCX 12 kb) [file 12891_2019_2465_MOESM2_ESM.docx]

Table S2. Underlying causes of death when rheumatoid arthritis/other musculoskeletal disorders were recorded as contributing cause of death.

| Contributing causes of death (ICD-10 codes) | Rheumatoid arthritis | Other musculoskeletal disorders |
| --- | --- | --- |
| Certain infectious and parasitic diseases (A00-B99) | 58 (5.4) | 137 (4.5) |
| Neoplasms (C00.0-D48.9) | 135 (12.5) | 451 (14.8) |
| Diseases of the blood and blood-forming organs (D50-D89) | 11 (1.0) | 25 (0.8) |
| Endocrine, nutritional and metabolic diseases (E00-E90) | 27 (2.5) | 122 (4.0) |
| Mental and behavioural disorders (F00-F99) | 34 (3.2) | 192 (6.3) |
| Diseases of the nervous system (G00-G99) | 28 (2.6) | 102 (3.3) |
| Diseases of the circulatory system (I00-I99) | 562 (52.1) | 1391 (45.5) |
| Diseases of the respiratory system (J00-J99) | 97 (9.0) | 255 (8.3) |
| Diseases of the digestive system (K00-K93) | 57 (5.3) | 114 (3.7) |
| Diseases of the genitourinary system (N00-N99) | 25 (2.3) | 69 (2.3) |
| Total death certificates | 1078 (100) | 3056 (100) |

Values are reported as number (%).
