# Supplementary material for: Educational inequalities in mortality associated with rheumatoid arthritis and other musculoskeletal disorders in Sweden
Source: BMC Musculoskelet Disord. 2019 Feb 18;20:83. doi: 10.1186/s12891-019-2465-8 (PMC6379941; doi:10.1186/s12891-019-2465-8)
Supplement: Supplementary file 3 — Table S3. Age-standardized mortality rate of rheumatoid arthritis and other musculoskeletal disorders by education group and sex. The Table reports age-standardized mortality rate related to rheumatoid arthritis and other musculoskeletal disorders and low to high education mortality rate ratio, stratified by sex. (DOCX 12 kb) [file 12891_2019_2465_MOESM3_ESM.docx]

Table S3. Age-standardized mortality rate of rheumatoid arthritis and other musculoskeletal disorders by education group and sex.

|  | Age-standardized mortality rates per 100,000 person-years | | | | Low education vs. high education | |
| --- | --- | --- | --- | --- | --- | --- |
|  | Overall | Low | Medium | High | Rate ratio (95% CI) | Rate difference/100,000 person-years |
| **Rheumatoid arthritis** |  |  |  |  |  |  |
| All | 12.0 (11.4, 12.7) | 14.1 (13.2, 15.1) | 10.8 (9.7, 12.0) | 6.4 (5.1, 7.9) | 2.2 (2.0, 2.4) | 7.8 |
| Men | 6.7 (5.9, 7.5) | 8.1 (7.0, 9.4) | 6.2 (4.9, 7.6) | 3.4 (2.2, 5.0) | 2.4 (2.0, 2.8) | 4.7 |
| Women | 15.7 (14.7, 16.6) | 17.8 (16.5, 19.2) | 14.4 (12.8, 16.1) | 9.2 (7.1 , 11.8) | 1.9 (1.7, 2.2) | 8.6 |
| **Other musculoskeletal disorders** |  |  |  |  |  |  |
| All | 33.0 (32.0, 34.1) | 36.4 (34.9, 38.0) | 31.9 (29.9, 33.9) | 24.3 (21.5, 27.3) | 1.5 (1.4, 1.6) | 12.2 |
| Men | 29.2 (27.6, 30.9) | 33.5 (31.1, 36.1) | 28.6 (25.8, 31.7) | 17.5 (14.2, 21.3) | 1.9 (1.7, 2.1) | 16.0 |
| Women | 35.0 (33.6, 36.4) | 38.0 (36.0, 40.0) | 33.7 (31.2, 36.4) | 30.2 (25.9, 35.0) | 1.3 (1.1, 1.4) | 7.7 |
